# Development of a Water Matrix Certified Reference Material for Volatile Organic Compounds Analysis in Water

**DOI:** 10.3390/molecules26144370

**Published:** 2021-07-20

**Authors:** Liping Fang, Linyan Huang, Gang Yang, Yang Jiang, Haiping Liu, Bingwen Lu, Yaxian Zhao, Wen Tian

**Affiliations:** State Environmental Protection Key Laboratory of Environmental Pollutant Metrology and Reference Materials, Institute for Environmental Reference Materials, Environmental Development Centre of the Ministry of Ecology and Environment, Beijing 100029, China; fang.liping@ierm.com.cn (L.F.); huang.linyan@ierm.com.cn (L.H.); yang.gang@ierm.com.cn (G.Y.); jiang.yang@ierm.com.cn (Y.J.); liu.haiping@ierm.com.cn (H.L.); lu.bingwen@ierm.com.cn (B.L.); zhao.yaxian@ierm.com.cn (Y.Z.)

**Keywords:** volatile organic compounds (VOCs), reference materials (RMs), quality control (QC), water analysis

## Abstract

Water matrix certified reference material (MCRM) of volatile organic compounds (VOCs) is used to provide quality assurance and quality control (QA/QC) during the analysis of VOCs in water. In this research, a water MCRM of 28 VOCs was developed using a “reconstitution” approach by adding VOCs spiking, methanol solution into pure water immediately prior to analysis. The VOCs spiking solution was prepared gravimetrically by dividing 28 VOCs into seven groups, then based on ISO Guide 35, using gas chromatography-mass spectrometry (GC-MS) to investigate the homogeneity and long-term stability. The studies of homogeneity and long-term stability indicated that the batch of VOCs spiking solution was homogeneous and stable at room temperature for at least 15 months. Moreover, the water MCRM of 28 VOCs was certified by a network of nine competent laboratories, and the certified values and expanded uncertainties of 28 VOCs ranged from 6.2 to 17 μg/L and 0.5 to 5.3 μg/L, respectively.

## 1. Introduction

Volatile organic compounds (VOCs), due to their toxicity and persistence in the environment, are one group of particularly important pollutants [1,2]. Many of these substances are toxic, and some are considered to be carcinogenic, mutagenic or teratogenic [3,4]. As reported, VOCs have been widely detected at trace levels in surface water and groundwater [2,5]. Therefore, during VOCs detection in water, references with VOCs are needed to calibrate the instrument, verify analysis procedure or control the analysis quality according to QA/QC (quality assurance and quality control) guidelines. Validation of the entire analytical procedure requires the use of an matrix certified reference material (MCRM), which is a homogeneous and well-defined matrix that contains known amounts of the target compounds [6,7,8]. However, there is currently no MCRM available for the measurement of VOCs in water. Therefore, in this research, we prepared and characterized a water MCRM of 28 VOCs.

There are several reasons for the current absence of MCRMs for organic materials in water. Specifically, the relatively low concentrations of most organic contaminants in water necessitates that large volumes of water be manipulated and transferred for analysis, which may lead to problems such as cross-contamination or improper and inaccurate dilution. Additionally, there is a tendency for target compounds to be adsorbed onto suspended or colloidal particles because of their low water solubility or their high lipophilic characteristics as expressed by the high octanol-water partition coefficient (*K*_ow_). Furthermore, the instability of some organic materials causes problems, as they may have a tendency to undergo hydrolysis, metabolization or photochemical reactions. Finally, the residual biological activity of samples can cause severe stability problems [9].

At present, the only commercially available water matrix reference material (MRM) is IRMM-428, released by the Institute for Reference Materials and Measurements (IRMM). IRMM-428 is prepared by spiking PFASs in methanol into a known volume of drinking water. Other attempts have been made to prepare proficiency testing (PT) samples for organics in water employing the widely used “reconstitution” approach. In this approach, a solution of analytes of interest in an organic solvent (miscible with water) is spiked into a water sample in the laboratory immediately prior to analysis [8,10,11] or at the producer’s premises just before shipping [12,13]. The rest of the approach is the ‘‘immobilization’’ of analytes (in this case, pesticides) on a solid-phase extraction (SPE) cartridge [8]. Water MRMs that consisted of pesticides stored on SPE cartridges were used in a collaborative study including 15 laboratories, and the observed reproducibility was 26.7% [10]. However, this approach is not sound from a metrological point of view because it was not possible to confirm that the true value equaled the initial concentration in the percolated water sample.

To the best of our knowledge, no water MRMs for VOCs are currently available for quality control. Therefore, in this research, we prepared and certified a water MCRM containing 28 VOCs. The water MCRM was prepared using a “reconstitution” approach by spiking the solution of VOCs in methanol into pure water in the laboratory immediately prior to analysis. The preparation and characterization of 28 VOCs spiking solutions were comprehensively studied, and the water MCRM containing all 28 VOCs was certified using a network of nine competent laboratories.

## 2. Results and Discussion

### 2.1. Methodology Study

**Figure 1:**
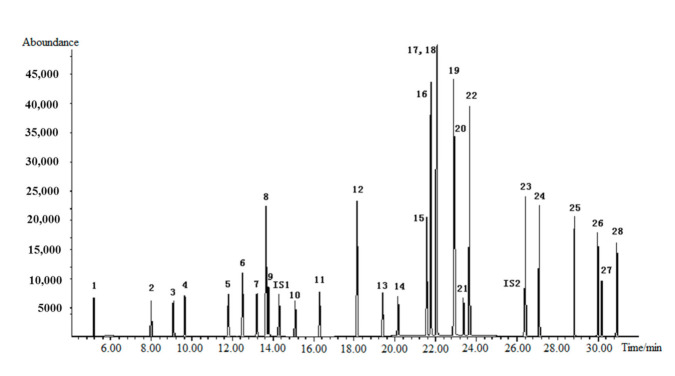
Total ion chromatography of 28 VOCs determined by GC-MS (1. vinyl chloride; 2. 1,1-dichloroethylene; 3. dichloromethane; 4. trans-1,2-dichloroethene; 5. cis-1,2-dichloroethene; 6. trichloromethane; 7. carbon tetrachloride; 8. benzene; 9. 1,2-dichloroethane; 10. trichloroethylene; 11. bromodicloromethane; 12. toluene; 13. tetrachloroethylene; 14. chlorodibromomethane; 15. chlorobenzene; 16. ethylbenzene; 17,18. p-xylene/m-xylene; 19. o-xylene; 20. styrene; 21. bromoform; 22. cumene; 23. 1,4-dichlorobenzene; 24. 1,2-dichlorobenzene; 25. 1,3,5-trichlorobenzene; 26. 1,2,4-trichlorobenzene; 27. hexachloro-1,3-butadiene; 28. 1,2,3-trichlorobenzene; IS1. fluorobenzene; IS2. 1,4-dichlorobenzene D4).

The 28 VOCs in the spiking solution were determined by GC-MS, and the total ion chromatography is shown in Figure 1. With the exception of p-xylene and m-xylene, 26 other VOCs, IS1 and IS2 were separated well on the chromatographic column.

The precision of the method was investigated by analyzing each reference material of 10 µg/mL 28 VOCs in methanol 10 times, and the results were presented in Table 1. The within laboratory RSDs of the method were between 0.14% and 2.55%, indicating good instrumental repeatability that could meet the requirements of homogeneity and stability studies. The detection limits of 28 VOCs in methanol were calculated based on three standard deviations, and the results were between 0.014 and 0.225 μg/mL. These levels were obviously lower than the concentrations of the 28 VOCs in the spiking solution, which ranged from 6.0 to 18 μg/mL. With the exception of hexachloro-1,3-butadiene, which had an *r* value of 0.9989, the linear correlation *r* values of the other 27 VOCs were higher than 0.9990, indicating good linear relationships.

### 2.2. Purity Test

The purity was used to correct the gravimetric preparation of the standard solutions, and therefore ensure the metrological traceability of the water MCRM. Detailed information regarding the manufacturer, labeled purities and the uncertainties of the 28 commercial standards are given in Table 1. The labeled purities of the commercial standards were verified by GC-FID using the peak area normalization method, and the results are presented in Table 1. The measured purities of all commercial standards were within the range of their labeled uncertainties. Therefore, the labeled purities and uncertainties were used during gravimetric preparation and calculation of the uncertainty in the characterization study of water MCRM, respectively, in consideration of the limitations of the peak area normalization method.

The purity test results of the batch of methanol showed that the methanol purity was acceptable. Additionally, no VOCs were detected, indicating that methanol was suitable for use as the solvent for the 28 VOCs spiking solution.

### 2.3. Homogeneity Assessment

Homogeneity is an important property of a reference material. Nevertheless, it is a relative concept closely related to the distribution of components in the material, sample size and the number of samples that have been selected to measure homogeneity [14].

**Figure 2:**
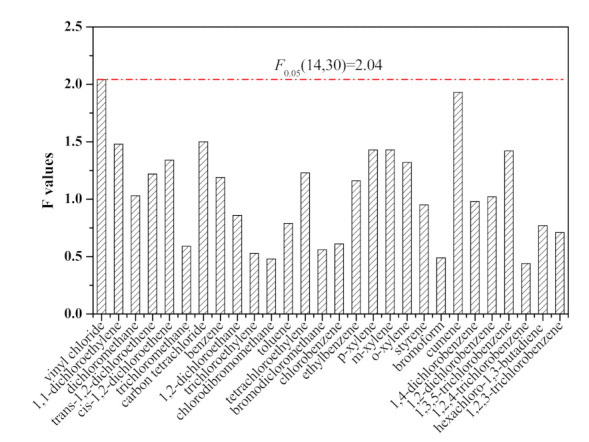
Results of homogeneity study of 28 VOCs in spiking solution.

In the present study, homogeneity was assessed by selecting 15 ampoules of the VOCs spiking solution using a stratified random samplings scheme covering the entire batch, after which three sub-samples of each ampoule were analyzed. The homogeneity study was evaluated using ANOVA [15], and the results are presented in Figure 2. All calculated *F* values were below or equal to the critical value *F*_0.05_(14,30) = 2.04, indicating no significant difference within bottles. The homogeneity analysis confirmed that the batch had a good agreement among its units (ampoules) for each of the 28 analytes in methanol, and the VOCs spiking solution was regarded as homogeneous.

It should be noted that the calculated *F* value of vinyl chloride was equal to the critical value *F*_0.05_(14, 30) = 2.04. The relatively high *F* value of vinyl chloride might relate to its high volatility and the relatively poor method repeatability using GC-MS, leading to the calculated MS_among_ being relatively higher than the MS_within_.

### 2.4. Stability Assessment

**Figure 3:**
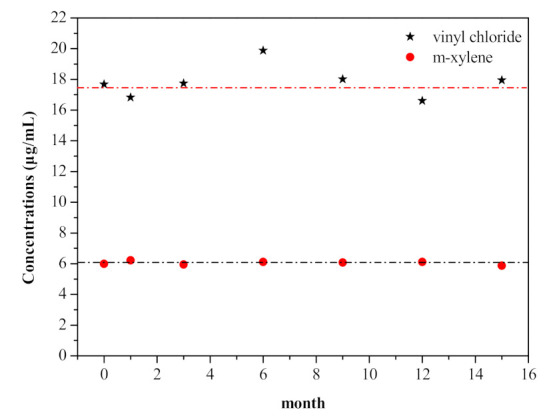
Results of vinyl chloride and m-xylene in spiking solution stored at room temperature at intervals of 15 months.

Stability testing is crucial to the certification of reference materials. The long-term stability study was based on linear regression [16], and the results measured after 0, 1, 3, 6, 9, 12 and 15 months of storage in different temperatures are shown in Figure 3 and Table 2, Table 3 and Table 4. At a confidence level of 95%, the observed slope for all results was |*b*_1_| < *t_0_*_.95,n-2_ × *s*(*b*_1_), indicating that the 28 analytes in the VOCs spiking solution stored at room temperature, 4 and −18 °C were stable after 15 months of storage. Therefore, the spiking solution, including the 28 VOCs, could be stored at room temperature for convenience, and the shelf-life of the VOCs spiking solution was at least 15 months.

Interestingly, the dispersions of the stability of the 28 VOCs under the same storage conditions were quite different. The stability of vinyl chloride and m-xylene after storage at room temperature for different lengths are shown as examples in Figure 3. The stability of vinyl chloride fluctuated with storage time around the preparation value in a relatively large range, although vinyl chloride in methanol was confirmed to be stable. However, the stability of m-xylene fluctuated closely around the preparation value with storage time. The obvious differences in stability results between vinyl chloride and m-xylene might be related to their characterizations. In the former methodology study, the within-laboratory RSD of vinyl chloride was 2.55%, while that of m-xylene was 0.43%. The lower boiling point of vinyl chloride than m-xylene might lead to significant differences in method precision [17], which could then lead to the different dispersions of stable results. Similar results were also observed for the other 26 VOCs, with the stable values of VOCs having higher boiling points generally being closer to their certified values than those having lower boiling points.

### 2.5. Characterization and Uncertainty Study

**Figure 4:**
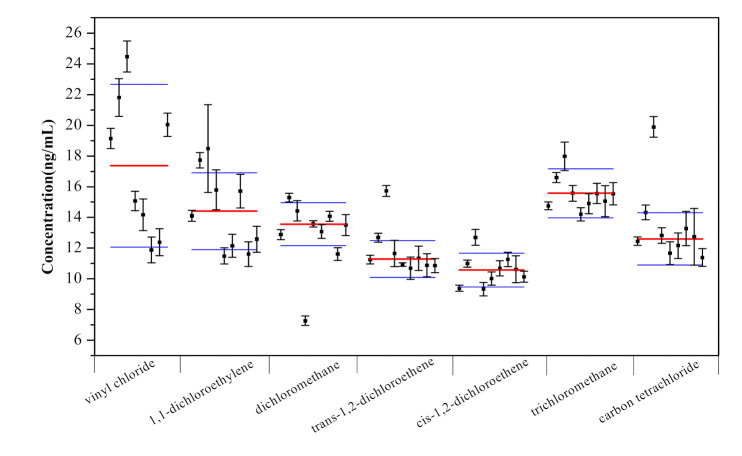

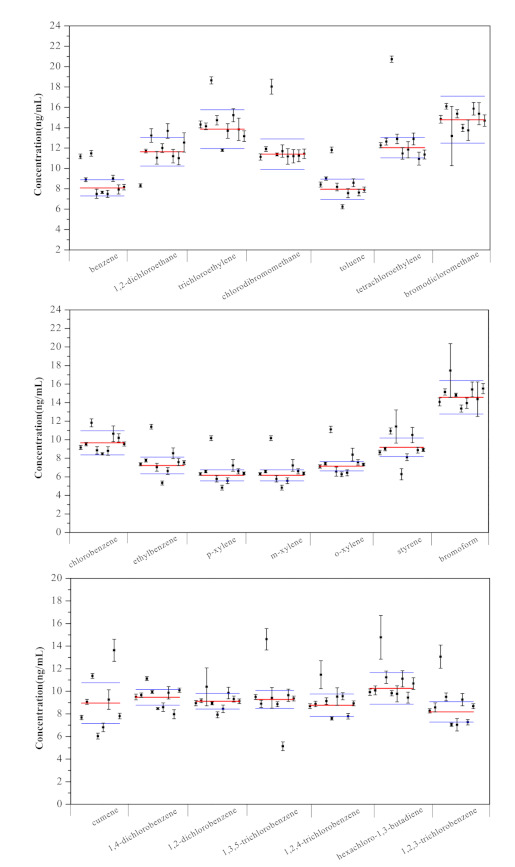
Measurement of 28 VOCs in water from nine laboratories. Thick red lines represent certified values, thin blue lines represent expended uncertainties.

The certification of the water MCRM was conducted by nine competent laboratories. Laboratory 4 used the headspace GC-MS method for certification, while the remaining eight laboratories used the purge-and-trap GC-MS method. The reported values of the 28 VOC analytes were expressed after diluting 1000 times in water. Statistical analysis was conducted for the received data using Grubb’s test, the Cochran test and Dixon’s test, and the mean values of the retained data were calculated as certified values. Figure 4 shows the reported mean values and standard deviations of the 28 VOCs in water from the nine laboratories. Most of the values were close to the certified values and within their expended uncertainties. However, some values from one or two laboratories showed obvious deviations from the certified values, such as the results reported from laboratory 3. Because both headspace GC-MS and purge-and-trap GC-MS are commonly used for the determination of VOCs in water, there were no obvious discrepancies in the results from the nine laboratories using the two different detection methods.

The results of the certified values and the expanded uncertainties of the water MCRM expressed at 95% confidence (with the coverage factor *k* = 2) are presented in Table 5. The certified values of the 28 VOCs in the water MCRM were in the range of 6.162 to 17.37 μg/L. The uncertainty of the water MCRM was calculated by combining the uncertainty of inhomogeneity (*u_bb_*), instability (*u_lst_*) and characterization (*u_char_*) [18], and the *u_bb_* and *u_lst_* were those of VOCs spiking solution. Some of the *u_bb_* were calculated through $u_{bb}=s_{bb}=\sqrt{\frac{MS_{\mathrm{among}}-MS_{\mathrm{within}}}{n}}$ when *MS*_among_ > *MS*_within_ for some VOCs, while others were calculated through $u_{bb}{}^{\prime }=\sqrt{\frac{MS_{\mathrm{within}}}{n}}\sqrt[{4}]{\frac{2}{v_{MS_{\mathrm{within}}}}}$ when *MS*_among_ < *MS*_within_. As shown in Table 5, the *u_bb_* of the 28 VOC analytes ranged from 0.18% to 3.73%, which was lower than the reported CRM of BTEX in methanol [19]. The *u_char_* and *u_lst_* of the 28 VOC analytes ranged from 0.87% to 9.44% and 0.91% to 11.4%, respectively. The expanded uncertainty of the 28 VOC analytes in the water MCRM ranged from 0.5 to 5.3 μg/L with a coverage factor *k* = 2 under an approximately 95% confidence level. The expanded uncertainty of o-xylene as 0.5 μg/L was lowest, and the expanded uncertainty of vinyl chloride as 5.3 μg/L was highest. The relatively high expanded uncertainty of vinyl chloride might result from two aspects. On the one hand, the highly volatile nature and early elution time on chromatography could result in a relatively high operation and instrument effect on quantification. On the other hand, the tested value of commercial vinyl chloride standard solution was not consistent with the labeled value, and large discrepancies existed among commercial standard solutions from different producers, making precise quantification difficult.

## 3. Materials and Methods

The Institute for Environmental Reference Materials, Ministry of Environmental Protection (IERM) has a quality management system based on ISO Guide 35 and ISO/IEC 17025, which is accredited by the China National Accreditation Service for Conformity Assessment (CNAS). The preparation and certification of the CRMs for environmental monitoring have been carried out according to the technical requirements of ISO Guide 35 [20].

### 3.1. Chemicals and Instruments

The commercial standards of 28 VOCs were purchased from several manufacturers. Detailed information regarding the manufacturers, purities and uncertainties of the purities are given in Table 6. Pesticide residue grade methanol was purchased from J.T. Baker, USA. The stock standard solutions were 27 mixed VOCs standard solution (IRMM, 100 µg/mL) and vinyl chloride standard solution (2000 µg/mL, Supelo, USA). The internal stock standard solutions were fluorobenzene and 1,4-dichlorobenzene D_4_ (IRMM, 1000 and 1000 µg/mL, respectively).

The VOCs spiking solution was prepared gravimetrically using a calibrated Mettler Toledo analytical balance (AE-240, 205 g capacity, resolution of 0.01 mg, Switzerland). The purities of the 28 VOC commercial standards were verified by a calibrated gas chromatography with flame ionization detection (Agilent 7890A GC-FID, USA). Homogeneity and stability studies of VOCs spiking solution were performed on a calibrated Agilent 7890A gas chromatograph coupled with an Agilent 5975C mass spectrometer (Agilent 7890A GC-5975C MS, USA). Analysis of the water MCRM was performed on a calibrated purge-and-trap Agilent 7890A GC-5975C MS.

### 3.2. Purity Test

The purities of the 28 VOC commercial standards were determined in-house using a GC-FID with a DB-1 column (30 m × 320 µm ID × 0.25 µm film). The oven temperature program started at 70 °C, then increased to 150 °C at 5 °C/min. The injection volume was 1 µL, and the injection was performed in split mode (30:1). The carrier gas was high purity nitrogen (1.0 mL/min), and the temperature of the injector and detector were 220 and 230 °C, respectively. The final purities of the 28 commercial standards were calculated by the peak area normalization method.

The purity of methanol (pesticides residue grade) selected as the solvent was checked by gas chromatography-mass spectrometry (GC-MS) prior to the preparation of CRM.

### 3.3. Determination of VOCs

#### 3.3.1. Determination of VOCs in the Spiking Solution

Measurement of the 28 VOCs in the spiking solution was performed on a GC-MS [21] equipped with a DB-624 (60 m × 250 µm ID × 1.4 µm film, Agilent, Santa Clara, CA, USA) capillary column. The oven temperature was programmed as follows: 35 °C for 2 min, followed by 5 °C/min to 120 °C, then 10 °C/min to 220 °C, where it was held for 3 min. The injection volume was 1 µL, and the injection was performed in split mode (30:1). The carrier gas was helium (1.0 mL/min), and the temperature of the injector, transfer line and ion source was 220, 260 and 230 °C, respectively. Data acquisition was performed under selected ion monitoring (SIM) mode.

#### 3.3.2. Determination of VOCs in Water MCRM

Analysis of the 28 VOCs in water MCRM was performed on a purge-and-trap GC-MS [21]. The conditions of the purge-and-trap were as follows: purge time: 11 min; purge rate: 40 mL/min; dry purge time: 1 min; desorption time: 2 min; desorption temperature: 190 °C; baking time: 6 min; baking temperature: 200 °C. The conditions of GC-MS were the same as for the determination of 28 VOCs in the spiking solution.

### 3.4. Preparation of the VOCs Spiking Solution

The 28 VOCs spiking solution was prepared gravimetrically. Briefly, the 28 VOCs were divided into seven groups during weighing and dissolution, then mixed into a certain volume. Among the 28 VOC commercial standards, only vinyl chloride standard is gaseous at room temperature. Therefore, vinyl chloride was placed in its own group, while the remaining 27 VOCs were divided into six groups according to their characteristics (Table 7). According to the labeled purities of the 28 VOC commercial standards, the stock solution of vinyl chloride in methanol was prepared by drawing a moderate volume of vinyl chloride using an airtight syringe and then adding it to methanol. For the other six groups, the stock solution of each group was prepared gravimetrically in the sequence of their polarity from weak to strong. After all stock solutions were prepared, a moderate volume of stock solution from each group was transferred into the same 1 L flask and then diluted to 1 L with methanol. The mass fractions of target compositions of the spiked solution were between 5.0 and 20 mg/L. Approximately 1 L of the VOC mixture was subdivided into 2 mL amber glass ampoules with 1.2 mL per ampoule using an ampoule filling machine. During the process of subdivision, 15 ampoules were sampled for a homogeneity study using a stratified random sampling strategy. After confirmation that the batch was homogeneous, the sealed ampoules were packed and divided into three parts, then stored at room temperature, 4 and −18 °C.

### 3.5. Homogeneity Testing

The VOCs spiking solution was subjected to a homogeneity study in which both the homogeneity between and within ampoules was evaluated. Fifteen ampoules of VOC spiking solution were selected using a stratified random sampling scheme covering the whole batch, and three sub-samples were analyzed in each ampoule. The internal standard was spiked into the solution for the QA/QC process, and each sample was analyzed in triplicate by GC-MS. Measurement sequences were randomized to be able to minimize possible trends in both the filling sequence and the analytical sequence.

The homogeneity was evaluated by one-way analysis of variance (ANOVA) as described by ISO Guide 35 [20]. The between-bottle standard deviation (*s_bb_*) and within-bottle standard deviation (*s_wb_*) were also calculated. Possible inhomogeneity was expressed as the uncertainty due to the between-bottle inhomogeneity of the material (*u_bb_*) and quantified as:

(1) $$u_{bb}=s_{bb}=\sqrt{\frac{MS_{\mathrm{among}}-MS_{\mathrm{within}}}{n}}$$

In cases in which *MS*_among_ < *MS*_within_ (indicating that the study set-up and/or method repeatability were not sufficient), the maximum heterogeneity that could be hidden by the method variability, the influence of analytical variation on the standard deviation between units *u_bb_*′ was calculated and used for estimates of in-homogeneity. The *u_bb_*′ was calculated as:

(2) $$u_{bb}{}^{\prime }=\sqrt{\frac{MS_{\mathrm{within}}}{n}}\sqrt[{4}]{\frac{2}{v_{MS_{\mathrm{within}}}}}$$

where *MS*_within_ is the mean square within groups determined from ANOVA, *n* is the number of replicates per bottle and $v_{MS_{\mathrm{within}}}$ represents the degrees of freedom of *MS*_within_.

The instrumental repeatability of the measurement of 28 VOCs was determined by conducting 10 replicate GC-MS analyses of 10 µg/mL reference material containing 28 VOCs.

### 3.6. Stability Testing

A long-term stability study was conducted to ensure the shelf-life of the VOCs spiking solution. A long-term stability study was conducted based on a classical stability study. Stability monitoring was performed for each analyte of the spiking solution after 0, 1, 3, 6, 9, 12 and 15 months of storage at room temperature, 4 and −18 °C. For each round of analysis, three ampoules were sampled randomly from those samples stored under each storage condition.

A linear regression model was utilized for processing data, namely assuming component values (*Y*) of time (*X*) varying linear equation as *Y* = *b*_0_ + *b*_1_*X*, where *b*_0_ and *b*_1_ are the regression coefficients. The estimated standard deviation of *b*_1_ is then given by:

(3) $$S(b_{1})=\frac{s}{\sqrt{\sum_{i=1}^{n}{\left(X_{i}-\bar{X}\right)}^{2}}}$$

where

(4) $$s^{2}=\frac{\sum_{i=1}^{n}{\left(Y_{i}-b_{0}-b_{1}X_{i}\right)}^{2}}{n-2}$$

and *t_cal_* is given by

(5) $$t_{cal}=\frac{\left|b_{1}\right|}{S\left(b_{1}\right)}$$

According to ISO Guide 35, the long-term instability is estimated as *u_lts_* = *S*(*b*_1_) × *t*, with *u_lts_* being the uncertainty of long-term instability, *S*(*b*_1_) is the standard error of the slope and *t* is the selected duration.

### 3.7. Characterization and Uncertainty Study

The certified values of 28 VOCs in the water MCRM were determined by the average of the results obtained from nine competent laboratories that each received six ampoules selected at random. Before analysis, the samples were diluted 1000 times with pure water, and the average concentrations of analytes after dilution were used.

Data sets were checked to ensure they followed approximately normal distributions and that variances for each compound were homogeneous. Grubb’s test was applied to evaluate within laboratory parallel data. Between-laboratory outliers of variance were detected using the Cochran test, while outliers of average were detected using Dixon’s test. Generally, the outliers detected from Grubb’s test were retained. When the RSD of within-laboratory parallel data was smaller than 15%, the stragglers and outliers from Cochran’s test were retained. The outliers (95% confidence interval) detected from Dixon’s test were removed.

The standard uncertainty of the certified values included collaborating characterization uncertainty (*u_char_*), between-bottle inhomogeneity uncertainty (*u_bb_*) and long-term instability uncertainty (*u_lts_*). The expanded uncertainty (*U*) of the certified property value was calculated as: $U=k\times \sqrt{u_{bb}{}^{2}+u_{lts}{}^{2}+u_{char}{}^{2}}$, where *k* is the coverage factor (usually set *k* = 2, approximately the 95% confidence level), *u_bb_* is the uncertainty due to inhomogeneity of the material, *u_its_* is the uncertainty due to instability of the material and *u_char_* is uncertainty in the characterization of the property value.

## 4. Conclusions

In conclusion, a water MCRM of 28 VOCs was developed using a “reconstitution” approach by adding the prepared VOCs spiking methanol solution into pure water directly prior to analysis. The 28 VOCs in methanol as a spiking solution was prepared gravimetrically by dividing the VOCs into seven groups. The batch of spiking solution was homogeneous and stable at room temperature, 4 and −18 °C for at least 15 months. The certification of the water MCRM was established in a study involving nine competent laboratories applying purge-and-trap GC-MS or headspace GC-MS. The certified values of the 28 VOC analytes in the MCRM ranged from 6.162 to 17.37 μg/L with expanded uncertainties in the range of 0.5 to 5.3 μg/L. The prepared water MCRM could be used for quality control during VOC analysis in water and for developing or verifying measurement methods for VOCs monitoring in water.

## Figures and Tables

**Table 1:** Precision and detection limits of 28 VOCs in methanol using GC-MS.

No.	Composition	RSD(%)	Detection Limit/ (μg/mL)	Linear Correlation *r*
1	vinyl chloride	2.55	0.1	1.000
2	1,1-dichloroethylene	1.53	0.1	0.9999
3	dichloromethane	0.78	0.07	1.000
4	*trans*-1,2-dichloroethene	1.03	0.07	0.9999
5	*cis*-1,2-dichloroethene	0.34	0.03	1.000
6	trichloromethane	0.26	0.03	1.000
7	carbon tetrachloride	0.74	0.04	0.9998
8	benzene	0.44	0.02	1.000
9	1,2-dichloroethane	0.50	0.04	1.000
10	trichloroethylene	0.72	0.2	1.000
11	bromodicloromethane	0.63	0.02	0.9998
12	toluene	0.53	0.03	0.9998
13	tetrachloroethylene	0.33	0.1	1.000
14	chlorodibromomethane	0.94	0.05	0.9997
15	chlorobenzene	0.45	0.02	0.9990
16	ethylbenzene	0.34	0.03	0.9999
17	p-xylene	0.43	0.04	0.9997
18	m-xylene	0.43	0.04	0.9997
19	o-xylene	0.43	0.03	0.9999
20	styrene	0.50	0.03	1.000
21	bromoform	0.39	0.03	0.9998
22	cumene	0.27	0.04	1.000
23	1,4-dichlorobenzene	0.18	0.01	0.9991
24	1,2-dichlorobenzene	0.14	0.02	0.9992
25	1,3,5-trichlorobenzene	0.27	0.02	0.9992
26	1,2,4-trichlorobenzene	0.28	0.01	0.9996
27	hexachloro-1,3-butadiene	0.50	0.06	0.9989
28	1,2,3-trichlorobenzene	0.31	0.02	0.9995

**Table 2:** Stability study of VOCs spiking solution stored at room temperature.

No.	Composition	*b*_0_ (μg/mL)	*b*_1_ (μg/mL)	*s*(*b*_1_) (μg/mL)	*t*_0.95,n−2_	*t_0_*_.95,n−2_ × *s*(*b*_1_)
1	vinyl chloride	18.04	−0.165	0.220	2.57	0.565
2	1,1-dichloroethylene	12.74	−0.048	0.080	2.57	0.207
3	dichloromethane	12.56	0.011	0.056	2.57	0.145
4	*trans*-1,2-dichloroethene	10.56	0.021	0.069	2.57	0.178
5	*cis*-1,2-dichloroethene	10.27	0.000	0.041	2.57	0.106
6	trichloromethane	13.72	0.112	0.160	2.57	0.410
7	carbon tetrachloride	12.00	0.069	0.074	2.57	0.190
8	benzene	8.654	−0.019	0.047	2.57	0.120
9	1,2-dichloroethane	10.83	0.059	0.067	2.57	0.172
10	trichloroethylene	14.00	0.049	0.109	2.57	0.281
11	chlorodibromomethane	14.49	0.117	0.100	2.57	0.257
12	toluene	8.580	−0.003	0.023	2.57	0.059
13	tetrachloroethylene	12.21	0.046	0.061	2.57	0.158
14	bromodicloromethane	12.09	0.051	0.109	2.57	0.280
15	chlorobenzene	9.873	−0.041	0.037	2.57	0.095
16	ethylbenzene	7.701	−0.028	0.020	2.57	0.050
17	p-xylene	6.092	−0.007	0.009	2.57	0.023
18	m-xylene	6.092	−0.007	0.009	2.57	0.023
19	o-xylene	7.191	−0.020	0.013	2.57	0.034
20	styrene	8.533	−0.021	0.017	2.57	0.043
21	bromoform	15.43	0.015	0.055	2.57	0.141
22	cumene	7.752	−0.010	0.005	2.57	0.012
23	1,4-dichlorobenzene	10.33	−0.053	0.029	2.57	0.074
24	1,2-dichlorobenzene	9.287	−0.032	0.026	2.57	0.066
25	1,3,5-trichlorobenzene	9.433	−0.019	0.029	2.57	0.073
26	1,2,4-trichlorobenzene	9.275	−0.040	0.040	2.57	0.102
27	hexachloro-1,3-butadiene	10.80	−0.040	0.057	2.57	0.147
28	1,2,3-trichlorobenzene	8.463	−0.006	0.025	2.57	0.063

**Table 3:** Stability study for VOCs spiking solution stored at 4 °C.

No.	Composition	*b*_0_ (μg/mL)	*b*_1_ (μg/mL)	*s*(*b*_1_) (μg/mL)	*t*_0.95,n−2_	*t_0_*_.95,n−2_ × *s*(*b*_1_)
1	vinyl chloride	17.06	0.108	0.158	2.57	0.405
2	1,1-dichloroethylene	12.11	0.059	0.050	2.57	0.128
3	dichloromethane	12.56	0.041	0.028	2.57	0.071
4	*trans*-1,2-dichloroethene	10.83	0.025	0.037	2.57	0.095
5	*cis*-1,2-dichloroethene	10.13	0.000	0.048	2.57	0.124
6	trichloromethane	14.77	0.048	0.044	2.57	0.113
7	carbon tetrachloride	12.11	0.079	0.041	2.57	0.105
8	benzene	8.330	0.010	0.017	2.57	0.045
9	1,2-dichloroethane	11.10	0.057	0.024	2.57	0.063
10	trichloroethylene	14.64	0.021	0.068	2.57	0.174
11	chlorodibromomethane	15.03	0.075	0.063	2.57	0.163
12	toluene	8.543	0.002	0.025	2.57	0.064
13	tetrachloroethylene	12.36	0.037	0.033	2.57	0.084
14	bromodicloromethane	11.83	0.073	0.092	2.57	0.238
15	chlorobenzene	10.03	−0.049	0.035	2.57	0.090
16	ethylbenzene	7.805	−0.032	0.020	2.57	0.050
17	p-xylene	6.156	−0.007	0.010	2.57	0.027
18	m-xylene	6.156	−0.007	0.010	2.57	0.027
19	o-xylene	7.259	−0.023	0.011	2.57	0.029
20	styrene	8.410	−0.009	0.011	2.57	0.028
21	bromoform	15.75	−0.023	0.041	2.57	0.107
22	cumene	7.753	−0.021	0.008	2.57	0.022
23	1,4-dichlorobenzene	10.21	−0.041	0.027	2.57	0.068
24	1,2-dichlorobenzene	9.324	−0.034	0.019	2.57	0.049
25	1,3,5-trichlorobenzene	9.432	−0.019	0.025	2.57	0.065
26	1,2,4-trichlorobenzene	9.275	−0.040	0.040	2.57	0.102
27	hexachloro-1,3-butadiene	10.28	−0.008	0.041	2.57	0.105
28	1,2,3-trichlorobenzene	8.578	−0.016	0.019	2.57	0.048

**Table 4:** Stability study for VOCs spiking solution stored at −18 °C.

No.	Composition	*b*_0_ (μg/mL)	*b*_1_ (μg/mL)	*s*(*b*_1_) (μg/mL)	*t*_0.95,n−2_	*t*_0.95,n−2_ × *s*(*b*_1_)
1	vinyl chloride	18.50	−0.018	0.139	2.57	0.358
2	1,1-dichloroethylene	12.48	0.021	0.052	2.57	0.134
3	dichloromethane	12.79	0.021	0.035	2.57	0.090
4	*trans*-1,2-dichloroethene	10.99	0.022	0.035	2.57	0.091
5	*cis*-1,2-dichloroethene	10.18	0.023	0.031	2.57	0.079
6	trichloromethane	14.82	0.051	0.043	2.57	0.110
7	carbon tetrachloride	12.21	0.073	0.049	2.57	0.126
8	benzene	8.434	0.011	0.021	2.57	0.054
9	1,2-dichloroethane	11.12	0.058	0.029	2.57	0.074
10	trichloroethylene	14.79	0.023	0.061	2.57	0.156
11	chlorodibromomethane	15.03	0.090	0.064	2.57	0.165
12	toluene	8.516	0.012	0.028	2.57	0.071
13	tetrachloroethylene	12.33	0.048	0.031	2.57	0.080
14	bromodicloromethane	11.61	0.091	0.060	2.57	0.154
15	chlorobenzene	10.093	−0.059	0.035	2.57	0.089
16	ethylbenzene	7.836	−0.034	0.020	2.57	0.051
17	p-xylene	6.168	0.001	0.011	2.57	0.028
18	m-xylene	6.168	0.001	0.011	2.57	0.028
19	o-xylene	7.243	−0.020	0.008	2.57	0.020
20	styrene	8.378	0.000	0.009	2.57	0.022
21	bromoform	15.64	−0.027	0.057	2.57	0.147
22	cumene	7.710	−0.012	0.007	2.57	0.017
23	1,4-dichlorobenzene	7.739	−0.021	0.006	2.57	0.015
24	1,2-dichlorobenzene	9.331	−0.032	0.018	2.57	0.047
25	1,3,5-trichlorobenzene	9.508	−0.032	0.022	2.57	0.057
26	1,2,4-trichlorobenzene	8.942	−0.018	0.023	2.57	0.060
27	hexachloro-1,3-butadiene	10.25	−0.013	0.042	2.57	0.107
28	1,2,3-trichlorobenzene	8.628	−0.028	0.016	2.57	0.042

**Table 5:** Certified values and expended uncertainties of 28 VOCs in the water MCRM.

No.	Composition	*u_char_* (%)	*u_bb_* (%)	*u_lst_* (%)	*Certified Value* (μg/L)	*u_CRM_* (*k* = 2) (μg/L)
1	vinyl chloride	9.44	3.73	11.4	1.7 × 10^1^	5.3
2	1,1-dichloroethylene	5.75	1.22	6.20	1.4 × 10^1^	2.5
3	dichloromethane	2.87	1.89	4.07	1.4 × 10^1^	1.4
4	*trans*-1,2-dichloroethene	2.04	0.84	4.75	1.1 × 10^1^	1.2
5	*cis*-1,2-dichloroethene	2.24	0.81	4.48	1.1 × 10^1^	1.1
6	trichloromethane	2.42	1.52	4.25	1.6 × 10^1^	1.6
7	carbon tetrachloride	2.61	1.71	5.80	1.3 × 10^1^	1.7
8	benzene	2.99	0.86	3.67	8.1 × 10^0^	0.8
9	1,2-dichloroethane	4.49	1.02	3.77	1.2 × 10^1^	1.4
10	trichloroethylene	2.68	1.20	6.09	1.4 × 10^1^	1.9
11	chlorodibromomethane	0.87	1.40	6.17	1.1 × 10^1^	1.5
12	toluene	3.74	0.46	4.81	7.9 × 10^0^	1.0
13	tetrachloroethylene	2.18	0.82	3.69	1.2 × 10^1^	1.0
14	bromodicloromethane	2.23	1.23	7.38	1.5 × 10^1^	2.3
15	chlorobenzene	3.65	0.50	5.38	9.7 × 10^0^	1.3
16	ethylbenzene	4.65	0.30	3.94	7.2 × 10^0^	0.9
17	p-xylene	4.25	0.31	2.62	6.2 × 10^0^	0.6
18	m-xylene	4.25	0.31	2.62	6.2 × 10^0^	0.6
19	o-xylene	3.46	0.31	1.64	7.1 × 10^0^	0.5
20	styrene	5.44	0.34	1.56	9.2 × 10^0^	1.0
21	bromoform	1.87	1.41	5.54	1.5 × 10^1^	1.8
22	cumene	9.87	0.42	1.32	9.0 × 10^0^	1.8
23	1,4-dichlorobenzene	3.15	0.18	0.91	9.5 × 10^0^	0.7
24	1,2-dichlorobenzene	1.74	0.22	3.00	9.1 × 10^0^	0.7
25	1,3,5-trichlorobenzene	1.56	0.25	3.60	9.3 × 10^0^	0.8
26	1,2,4-trichlorobenzene	3.76	0.39	3.98	8.8 × 10^0^	1.0
27	hexachloro-1,3-butadiene	1.83	0.77	6.14	1.0 × 10^1^	1.4
28	1,2,3-trichlorobenzene	4.72	0.31	2.91	8.2 × 10^0^	0.9

**Table 6:** Purities of 28 commercial VOC standards.

No.	Composition	Manufacturer	Labeled Purity (%)	Labeled Uncertainty (%)	Measured Purity (%)
1	vinyl chloride	gmgas, China	99.999	0.5	99.99
2	1,1-dichloroethylene	ChemService,USA	99.5	0.5	99.83
3	dichloromethane	ChemService,USA	99.5	0.5	99.74
4	*trans*-1,2-dichloroethene	ChemService,USA	99.3	0.5	99.37
5	*cis*-1,2-dichloroethene	ChemService,USA	99.5	0.5	99.78
6	trichloromethane	ChemService,USA	99.5	0.5	99.34
7	carbon tetrachloride	ChemService,USA	99.5	0.5	99.85
8	benzene	ChemService,USA	99.5	0.5	99.95
9	1,2-dichloroethane	ChemService,USA	99.5	0.5	99.93
10	trichloroethylene	ChemService,USA	99.5	0.5	99.83
11	chlorodibromomethane	Fluka,USA	98.8	0.5	98.88
12	toluene	ChemService,USA	99.5	0.5	99.99
13	tetrachloroethylene	ChemService,USA	99.5	0.5	99.94
14	bromodicloromethane	Fluka,USA	99.5	0.5	99.86
15	chlorobenzene	ChemService,USA	99.5	0.5	99.91
16	ethylbenzene	ChemService,USA	99.5	0.5	99.48
17	p-xylene	ChemService,USA,USA	99.5	0.5	99.77
18	m-xylene	ChemService,USA	99.4	0.5	99.83
19	o-xylene	ChemService,USA	99.0	0.5	99.27
20	styrene	ChemService,USA	99.4	0.5	99.86
21	bromoform	ChemService,USA	99.5	0.5	99.53
22	cumene	ChemService,USA	99.5	0.5	99.93
23	hexachloro-1,3-butadiene	AccuStandard,USA	98.3	1.0	97.82
24	1,4-dichlorobenzene	ChemService,USA	99.5	0.5	99.98
25	1,2-dichlorobenzene	ChemService,USA	99.5	0.5	99.73
26	1,2,4-trichlorobenzene	ChemService,USA	99.5	0.5	99.58
27	1,2,3-trichlorobenzene	ChemService,USA	99.5	0.5	99.93
28	1,3,5-trichlorobenzene	ChemService,USA	99.5	0.5	99.11

**Table 7:** Grouping of 28 VOCs during preparation of VOCs spiking solution.

Group	Composition
1	vinyl chloride
2	dichloromethane, trichloroethylene, bromoform, chlorodibromomethane
3	carbon tetrachloride, bromodicloromethane, tetrachloroethylene
4	trichloromethane, 1,2-dichloroethane, 1,4-dichlorobenzene, hexachloro-1,3-butadiene
5	toluene, ethylbenzene, o-xylene, m-xylene, p-xylene, styrene, cumene
6	chlorobenzene, 1,2-dichlorobenzene, 1,2,4-trichlorobenzene, 1,2,3-trichlorobenzene, 1,3,5-trichlorobenzene
7	1,1-dichloroethylene, benzene, trans-1,2-dichloroethene, cis-1,2-dichloroethene
